# How are nurses’ individual and their colleagues’ diversity factors associated with their cooperation behaviors in teams? - Results of an online factorial survey

**DOI:** 10.1186/s12912-026-04572-5

**Published:** 2026-03-24

**Authors:** Anna Schneider, Anna Maria Khouri, Lisa Peppler, Martin Feißt, Robert Roehle, Daniel Drewniak, Liane Schenk

**Affiliations:** 1 Institute of Medical Sociology and Rehabilitation Science, Charité – Universitätsmedizin Berlin, corporate member of Freie Universität Berlin and Humboldt-Universität zu Berlin, Charitéplatz 1, 10117 Berlin, Germany; 2 Metaplan - Gesellschaft für Planung und Organisation mbH, Goethestraße 16, 25451 Quickborn, Germany; 3 Institute of Biometry and Clinical Epidemiology, Charité – Universitätsmedizin Berlin, corporate member of Freie Universität Berlin and Humboldt-Universität zu Berlin, Charitéplatz 1, 10117 Berlin, Germany; 4 Clinical Trial Office, Charité – Universitätsmedizin Berlin, corporate member of Freie Universität Berlin and Humboldt-Universität zu Berlin, Charitéplatz 1, 10117 Berlin, Germany; 5 Berlin Institute of Health at Charité – Universitätsmedizin Berlin, Anna-Louisa-Karsch-Str. 2, 10178 Berlin, Germany; 6 Institute of Biomedical Ethics and History of Medicine, University of Zürich, Zurich, Switzerland

**Keywords:** Nurse, Team, Diversity, Cooperation, Vignette, Factorial survey

## Abstract

**Background:**

Diversity within nursing teams in Germany increases due to staff shortages, nurse migration and the current academization of the profession. Little is known about how nurse’s own diversity factors and those of team members impact nurse’s perception and behavior in team situations. This study examines if and how nurses differentiate in the assessment of their colleagues’ nursing abilities in basic care, i.e. patient’s personal hygiene and treatment care, i.e. the set-up of an infusion, as well as their willingness to swap shifts with colleagues.

**Methods:**

A quasi-experimental, cross-sectional study with a factorial online survey with fictional case studies (vignettes) was conducted at two healthcare organizations in 2023 in a major city in Germany. Nurses ( > = 18 years) working in hospitals or nursing homes were eligible to participate. Associations of diversity factors, i.e., gender, age, migration background, and qualification, on the participant and vignette level on perceived competences and willingness to cooperate were analysed via multi-level fixed effects models.

**Results:**

*N* = 684 nurses participated (76% female; mean age: 44.2 years; 88% born in Germany; 75% completed vocational training). Colleagues’ competency in patient’s personal hygiene was rated higher by respondents born in Germany (β = 0.42, *p*=.002; vs. foreign-born), when fictional colleagues were from Germany (β = 0.26, *p*<.001) or France (β = 0.09, *p*=.01; vs. China) and had completed vocational training (β = 0.06, *p*=.02; vs. a university degree). Competency in inserting infusions was rated higher by respondents born in Germany (β = 0.59, *p*=.002; vs. foreign-born), when fictional colleagues were from Germany (β = 0.33, *p*<.001) or France (β = 0.21, *p*<.001; vs. China) and when one worked together for months (β=-0.44, *p*<.001; vs. days). Willingness to swap shifts was affected by the length of collaboration (β=-0.09, *p*<.001) and their fictional colleagues’ age (β=-0.07, *p*=.02).

**Conclusions:**

How nurses perceive their colleagues’ competences tended to be associated with their own and the other person’s migration background, while willingness to cooperate was affected by length of collaboration and colleagues’ age. However, limited generalizability of the findings applies due to our highly selective sample. Improving the onboarding process of new nurses in teams, and providing the time and space for professional exchange at work might improve mutual trust and teamwork in nursing.

**Clinical trial number:**

Not applicable.

**Supplementary Information:**

The online version contains supplementary material available at 10.1186/s12912-026-04572-5.

## Background

Nursing teams in Germany are characterized by an increasing diversity of their team members, partly due to the continuous migration of professionals in the course of targeted recruitment from abroad and the increasing number of nurses with academic professional qualifications acquired at German or foreign universities [1]. Challenges in the workplace faced by these nurses from foreign countries include language barriers with patients and colleagues, familiarity with the German healthcare system and the scope of nursing activities as well as a potentially different self-image of nursing staff [2]. The proportion of women among all nursing staff in Germany remains disproportionately high with 82% in 2024, but the workforce is continuously diversifying with regard to gender and country of origin. 16% of nurses had a foreign citizenship in 2024, however, this percentage is forecasted to increase, such as the share of male nurses, also due to the entry of refugees from countries outside the European Union (EU) into the nursing labour market [1].

Since healthcare provision is highly specialized and complex, inter- and intraprofessional teamwork is essential in providing effective care to patients with varying needs. Among other factors, the increasing diversity of nurses affects teamwork in healthcare organizations and ultimately impacts the quality of care provision and patient outcomes [3, 4]. However, research on nurses’ individual implications for practice regarding their colleagues’ diversity factors is scarce. While previous research has mostly focused on repercussions for individual nurses from working in diverse nursing teams, this quantitative study is novel in its focus on the simultaneous consideration of different diversity dimensions (gender, age, country of origin, qualification) and their direct impact on the perception of team members’ nursing competences using a factorial survey design. This approach allows for an analysis of the differential impact of different diversity variables measured on the level of the survey respondent and the level of (fictitious) team members on nurses’ individual perceptions.

### Conceptual framework

The term ‘diversity’ in our study encompasses individuals’ social differentiations with respect to their country of birth, migration experience, age and gender as well as their qualification in nursing. In the broader context, sociological and psychological research works with different concepts which are often embedded in the discourse on diversity management, namely prejudices, stereotypes and discrimination. Prejudices are described as mostly negative attitudes towards a specific group and their members with the aim of “creat[ing] or maintain[ing] hierarchical status relations between groups” [5]. Stereotypes, however, consist of specific characteristics which are assumed to represent “the essence of a group”, and which in turn influence how a person perceives, interprets and reacts to respective members of this group. They are received and passed on through one’s social environment, the media, and conversational patterns [5].

Both, prejudices and stereotypes can result in discriminatory behavior towards specific groups. In this regard, discrimination exercised by individuals, describes efforts to privilege the members of one specific group over the members of another [5]. These considerations touch upon the assertions of Tajfel’s Social Identity Theory (SIT) [6], according to which one’s perceived positive social identity, which is determined by the perceived membership in one or various groups marked by different social differentiations, influences the perception and moral judgment of other individuals outside one’s own in-group. The related concept of in-group favouritism describes individuals’ inclination to perceive and evaluate the actions and behaviors of members of one’s in-group more positively than those of members of the out-group [7]. Drawing on SIT, nurses in our sample might categorize colleagues into ‘in-groups’ and ‘out-groups’ based on diversity characteristics such as age, gender, country of origin and qualification. Accordingly, nurses might display an in-group favoritism, where the competence of colleagues with similar diversity characteristics is rated higher to maintain a positive social identity with the in-group. Similarly, willingness to cooperate might be stronger with members from the perceived in-group.

However, apart from individual attitudes and behavioral patterns, familiarity between team members in healthcare settings can impact intra- and interprofessional team work. Thus, on wards with higher resident-to-nurse familiarity, nurses evaluated relationships between team members more favorable than on wards with lower resident-to-nurse familiarity [8]. Drawing on Allport’s intergroup contact theory, prolonged interpersonal contact between individuals might reduce the reliance on stereotypes and resulting prejudices and discrimination [9]. Especially under conditions of the necessity to pursuit common goals and to cooperate along the way, prejudices are likely to be reduced. As nurses interact over a longer period of time in everyday work, initial category-based perceptions might be replaced by individuated trust in colleagues’ nursing competences, and thus willingness to cooperate might increase. However, existing research on nurse samples differentiated by their migration status shows mixed effects on the associations of prejudice and the length of interpersonal contact [10]. Overall, everyday contact between nurses with and without migration experience often fails to meet the favorable conditions of intergroup contact theory and therefore does not reliably reduce prejudice. In several contexts it is actually associated with ongoing discrimination and marginalization, while a few studies demonstrate that high-quality, cooperative contact under supportive conditions can improve attitudes and team relations [10].

Using a factorial survey design, our study examines if and how nurses differentiate in the assessment of their colleagues’ nursing abilities depending on several social characteristics that do not primarily relate to these colleagues’ actual abilities, e.g., their age, gender, country of birth, and qualification. Furthermore, our study examines if nurses’ own diversity characteristics and the length of collaboration with colleagues are associated with the assessment described above.

## Methods

### Design and setting of the study

This study was part of the mixed-methods research project Care in Transition (CareTrans) conducted between September 2021 and December 2024. CareTrans addressed changes in processes, structures and organizational cultures linked to the increasing diversity of nursing teams in hospitals and nursing homes and respective implications for care provision. Furthermore, the project aimed to optimize organizational learning from dealing with diversity in healthcare by developing instruments for organizational and personnel development in a participatory approach.

Currently, nursing education in Germany can be pursued through two main pathways. One long-established option is the completion of a vocational training program. Admission prerequisites generally include an intermediate secondary school certificate (*Realschulabschluss*) or another form of general education comprising ten years of schooling, or alternatively, a lower secondary school certificate (*Hauptschulabschluss*, nine years of schooling) combined with previously completed training as a nursing assistant or care aide. In addition, applicants must provide evidence of sufficient proficiency in the German language. The generalist nursing education program in Germany extends over a period of three years on a full-time basis. The curriculum encompasses both theoretical instructions delivered at accredited nursing schools and extensive practical training within healthcare institutions, such as hospitals, inpatient or long-term care facilities, and home-based nursing services. The second option consists of a nursing degree program of at least three years duration, for which the *Abitur* (general higher education entrance qualification) represents the most common admission requirement. This program likewise follows a generalist orientation and integrates both theoretical and practical components, with a strong emphasis on clinical placements across institutions providing acute and long-term care in both inpatient and outpatient settings.

Our online survey was conducted between May and August 2023 at two healthcare organizations which operate hospitals and nursing homes in a major city in Germany. Study results are presented without naming the specific organizations based on the procedure specified in the research proposal. The two healthcare organizations were chosen due to their size, numbers of employees and one institution due to ease of survey implementation. All nurses of legal age and active in healthcare provision at the time of the survey were eligible to participate. Nurses were recruited through multiple emails to their work addresses containing an invitation to participate and a corresponding link to the survey. Furthermore, printed flyers with a short information on the survey and a quick-response (QR) code were distributed in participating hospitals and nursing homes. Lastly, study announcements were published on the intranet sites of both institutions. The online survey was administered with the web-based survey software Unipark. All study materials were translated into English by a professional interpreter. Participation in the survey was voluntary. No compensation was granted. Two reminders were sent out two weeks and one month apart via email and with intranet announcements.

### Study methods

A factorial survey is a quasi-experimental method used to study complex social situations [11], which is increasingly used for research in healthcare settings [12, 13]. Study participants are confronted with different hypothetical scenarios, i.e., vignettes. The vignettes describe one specific scenario, however, several factors in the respective scenario are experimentally varied between vignettes. Study participants are asked to put themselves in the described situation and to answer one or more questions regarding the scenario after reading each individual vignette. Using this approach, the simultaneous associations of several factors on one outcome can be ascertained [14]. No standardized sample size calculation procedures for factorial surveys are established yet [11]. However, due to the hierarchical data structure of the vignette design, a sufficient number of cases at vignette level can be expected if the sets are designed accordingly. This allows robust statements to be made regarding the parameters to be estimated when using multi-level analyses [15, 16]. No prior sample size calculations were performed before the analysis of the explorative research question of this paper.

The vignettes and outcomes used in this study were developed in a multistage process from the first qualitative data of the project, i.e., participant observation protocols, interview and focus group material, and a literature review on conflict situations in nursing teams. Three different scenarios were developed through multiple focus discussions within the project team. Cursory content analysis of available qualitative data from the mixed methods research project and reconciliation with the results of available empirical studies and other topical nursing literature was incorporated (see Supplemental Material 1). Variables were selected with regard to their significance for the current discourse on the impact of diversity in German nursing teams set forth by the focus of the overall project CareTrans (age, gender, country of birth, qualification). A pre-test of the vignettes was conducted including qualitative interviews with nurses and social science researchers and an online survey at two wards of a participating hospital.

Data from factorial surveys is hierarchically structured on the respondent level and the vignette level. The total amount of vignettes in one scenario is made from all possible and logical combinations of the included varied factors. In our study, first, the main effects for the regression equations in the three individual scenarios were determined, followed by the calculation of the entire vignette universe for each scenario. There were no illogical combinations in the vignette universe. Using a macro in the statistical software SAS, sets were created from the entire vignette universes. A d efficiency of 100 per scenario was calculated. Each participant was randomly assigned a set of three vignettes per scenario during the survey. The order of the vignettes within the individual sets was also randomized to avoid potential sequence effects.

Three fictional nursing scenarios were implemented. In these scenarios, respondents were asked to put themselves in a situation in which they were asked to (a) take on basic (patient’s personal hygiene) or (b) treatment (infusion) nursing activities in collaboration with a colleague or (c) to swap duties with a colleague. Dimensions and variants are described in Table 1. Regarding the dimension ‘country of origin’, France and China were chosen as variants due to two main reasons: first, in both countries, there are (or have been) two educational pathways that lead to a career in nursing, i.e., those with a stronger focus on vocational nursing training as well as academic nursing degree programs. Thus, all three countries (Germany, China, France) meet the requirement that all variants of the dimension ‘qualification’ are valid and realistic for all three countries in the factorial survey (in comparison to other countries where nursing education is regulated via one specific educational pathway). Second, both countries act as proxies and are intended to be applied interchangeably. They primarily reflect the socio-cultural (concerning the social system and associated values and norms) and geographical proximity to certain countries as potentially perceived by a large number of individuals in the German context. Only scenarios (b) and (c) included the dimension “length of collaboration”. This resulted in a total of 36 different vignettes with varying characteristics for the ‘personal hygiene’ scenario and 72 different vignettes each for the ‘infusion’ and ‘swap duty’ scenarios. The endpoints of interest were operationalized as the participants’ perceived confidence in the abilities of the fictitious colleagues in the respective scenarios. They were measured after each individual vignette on a six-point Likert scale with one item from the self-rating perspective and one item from the colleague-rating perspective. The latter asked participants to evaluate the scenario from their presumed actual colleagues’ point of view in order to reduce the effects of potential social desirability bias in response behavior. In addition to the factorial survey, the online survey included items on socio-demographic and professional characteristics of the participants. Relevant variables regarding participants’ own diversity factors for this analysis included age, gender, migration experience and professional qualification.

**Table 1:** List of dimensions and variants in the factorial survey

Dimension	Variants
Gender	Male Female
Age	24 years 42 years 59 years
Country of origin	Germany France China
Qualification	Nursing degree Nursing training
Length of collaboration	A few days A few months

### Statistical analyses

The hierarchical data structure was analysed using linear mixed models with random intercepts in IBM SPSS Statistics (version 29.0). Diversity characteristics of the participating nurses (age, gender, migration experience, qualification) were used as regressors on the macro level, and the varying diversity characteristics of the fictitious colleagues in vignettes were used as regressors on the micro level. A random intercept model was carried out separately for each of the three scenarios and for each outcome measured within the vignettes, i.e., participants’ assessment from their own perspective and from the perspective of their actual colleagues. This resulted in six different models (three scenarios with two outcomes each). All participants who answered at least one of the respective vignettes served as the analysis population for the ‘personal hygiene’ and ‘swap duty’ scenarios. Using filters, the ‘infusion’ scenario was only administered to participants with at least three years of professional training, as less qualified nursing staff are not authorized to carry out this activity independently in Germany. Results from the study are to be interpreted in an exploratory manner.

## Results

A total of *n* = 684 nurses took part in the online survey. Participants with complete information identified as female (*n* = 413; 75.6%), male (*n* = 126; 23.1%) or diverse (*n* = 7; 1.3%). They were on average 44.2 (SD: 11.6) years old. Most reported three years of vocational training (75.2%), while 17.6% held a university degree related to nursing and 4.3% had one- to two-year vocational training. The majority of respondents stated Germany as their country of birth (88.2%).

### Case scenario 1: patient’s personal hygiene

The case scenario ‘personal hygiene’ was answered by *n* = 523 nurses. German-born respondents tended to have a higher level of confidence in the competence of their fictitious colleagues in terms of their abilities to conduct patient’s personal hygiene activities compared to foreign-born respondents (β = 0.42, 95% CI: 0.15 to 0.69, *p*=.002; see Table 2). If the fictitious colleagues were of German (β = 0.26, 95% CI: 0.19 to 0.32, *p*<.001) or French origin (β = 0.09, 95% CI: 0.02 to 0.15, *p*=.01), nurses trusted them more with personal hygiene activities than fictitious colleagues from China. Qualifications had a relatively small effect: Fictitious colleagues who held a university degree were less trusted by nursing staff when it came to personal hygiene than fictitious colleagues with vocational nursing training (β=-0.06, 95% CI: -0.12 to -0.01, *p*=.02). When comparing these results with respondents’ answers from their colleagues’ perspective, all but one association were similar in effect size and p-level. When answering the vignettes from their colleagues’ point of view, the trust in studied fictitious nurses in comparison to vocationally trained nurses with regard to personal hygiene was not significantly different (see Supplemental Material 2). 

**Table 2:** Results of the random intercept model on the outcome ‘confidence in colleagues’ competencies regarding patient’s personal hygiene’

Parameter	β [95% CI]	SD	df	t	*p* value
Constant term	4.76 [4.39; 5.13]	0.19	475.66	25.21	< 0.001
Participant variables (macro level)					
Gender (reference: female)					
Male	0.07 [-0.12; 0.26]	0.10	448.45	0.69	0.49
Diverse	0.48 [-0.28; 1.25]	0.39	447.69	1.24	0.22
Age	0.00 [-0.01; 0.01]	0.00	449.34	0.64	0.52
Birth country (reference: outside Germany)					
Germany	0.42 [0.15; 0.69]	0.14	449.43	3.05	0.002
Qualification (reference: nurse graduate)					
Nursing assistant	-0.42 [-0.85; 0.01]	0.22	448.08	-1.93	0.06
Registered nurse	-0.20 [-0.42; 0.01]	0.11	449.58	-1.84	0.07
Vignette variables (micro level)					
Age (reference: 59 years)					
24 years	-0.03 [-0.10; 0.03]	0.03	893.00	-1.03	0.30
42 years	0.06 [-0.01; 0.12]	0.03	894.48	1.77	0.08
Gender (reference: female)					
Male	-0.01 [-0.06; 0.05]	0.03	916.74	-0.20	0.85
Country of origin (reference: China)					
Germany	0.26 [0.19; 0.32]	0.03	893.48	7.95	< 0.001
France	0.09 [0.02; 0.15]	0.03	893.33	2.71	0.01
Qualification (reference: vocational training)					
Graduate degree	-0.06 [-0.12; -0.01]	0.03	916.24	-2.29	0.02

Note: β standardized regression coefficient; CI confidence interval; SD standard deviation; df degrees of freedom; t test statistic t; p probability

### Case scenario 2: starting an infusion

The case scenario ‘infusion’ was answered by *n* = 460 nurses. Respondents born in Germany tended to have a higher level of confidence in the competence of their fictitious colleagues when setting up an infusion than nurses born abroad (β = 0.59, 95% CI: 0.22 to 0.95, *p*=.002; see Table 3). If fictitious colleagues were of German (β = 0.33, 95% CI: 0.22 to 0.43, *p*<.001) or French origin (β = 0.21, 95% CI: 0.10 to 0.31, *p*<.001), nurses trusted them more to administer an infusion than fictitious colleagues from China. Fictitious colleagues with whom nurses have only been working for a few days were trusted less to set up an infusion than fictitious colleagues with whom they have been working for months (β=-0.44, 95% CI: -0.53 to -0.35, *p*<.001). These results mostly applied to the participants’ self-report as well as to their assessment from their colleagues’ perspective. However, nurses born abroad did not differ from nurses born in Germany when answering the vignettes from their colleagues’ point of view (see Supplemental Material 3). 

**Table 3:** Results of the random intercept model on the outcome ‘confidence in colleagues’ competencies regarding infusion insertion’

Parameter	β [95% CI]	SD	df	t	*p* value
Constant term	4.57 [4.06; 5.08]	0.26	462.75	17.52	< 0.001
Participant variables (macro level)					
Gender (reference: female)					
Male	0.17 [-0.09; 0.44]	0.13	418.55	1.28	0.20
Diverse	0.74 [-0.27; 1.74]	0.51	413.44	1.44	0.15
Age	-0.01 [-0.02; 0.00]	0.01	417.72	-1.12	0.26
Birth country (reference: outside Germany)					
Germany	0.59 [0.22; 0.95]	0.19	413.60	3.13	0.002
Qualification (reference: nurse graduate)					
Registered nurse	-0.12 [-0.42; 0.18]	0.15	413.92	-0.79	0.43
Vignette variables (micro level)					
Age (reference: 59 years)					
24 years	-0.01 [-0.12; 0.09]	0.05	808.05	-0.27	0.79
42 years	-0.08 [-0.18; 0.03]	0.05	808.96	-1.41	0.16
Gender (reference: female)					
Male	-0.05 [-0.14; 0.04]	0.05	839.87	-1.03	0.30
Country of origin (reference: China)					
Germany	0.33 [0.22; 0.43]	0.05	808.96	6.08	< 0.001
France	0.21 [0.10; 0.31]	0.05	808.14	3.91	< 0.001
Qualification (reference: vocational training)					
Graduate degree	0.02 [-0.07; 0.11]	0.05	839.91	0.36	0.72
Length of cooperation (reference: a few months)					
A few days	-0.44 [-0.53; -0.35]	0.05	839.39	-9.55	< 0.001

Note: β standardized regression coefficient; CI confidence interval; SD standard deviation; df degrees of freedom; t test statistic t; p probability

### Case scenario 3: swapping shifts

A total of *n* = 481 nurses answered the case scenario regarding their willingness to swap shifts with colleagues. Observed effect sizes in this scenario were relatively small in comparison to the above described scenarios. Participants were less willing to swap shifts with fictitious colleagues with whom they have only been working for a few days than for a few months (β=-0.09, 95% CI: -0.14 to -0.04, *p*<.001, see Table 4). In addition, participants would have rather swapped with older colleagues than with middle-aged colleagues (β=-0.07, 95% CI: -0.13 to -0.01, *p*=.02). Nurses also stated that their actual colleagues were more likely to swap shifts with fictitious colleagues from Germany (β = 0.11, 95% CI: 0.05 to 0.18, *p*<.001) than with fictitious colleagues from China (see Supplemental Material 4).

**Table 4:** Results of the random intercept model on the outcome ‘willingness to swap shifts’

Parameter	β [95% CI]	SD	df	t	*p* value
Constant term	5.12 [4.69; 5.54]	0.22	451.45	23.41	< 0.001
Participant variables (macro level)					
Gender (reference: female)					
Male	-0.05 [-0.28; 0.17]	0.11	429.71	-0.46	0.65
Diverse	0.09 [-0.77; 0.95]	0.44	428.73	0.21	0.84
Age	0.00 [-0.01; 0.01]	0.00	430.62	-0.09	0.93
Birth country (reference: outside Germany)					
Germany	0.27 [-0.04; 0.58]	0.16	435.68	1.71	0.09
Qualification (reference: nurse graduate)					
Nursing assistant	-0.68 [-2.06; 0.70]	0.70	428.83	-0.97	0.33
Registered nurse	-0.22 [-0.47; 0.03]	0.13	431.45	-1.70	0.09
Vignette variables (micro level)					
Age (reference: 59 years)					
24 years	0.00 [-0.06; 0.06]	0.03	849.73	-0.09	0.93
42 years	-0.07 [-0.13; -0.01]	0.03	850.09	-2.35	0.02
Gender (reference: female)					
Male	0.04 [-0.02; 0.09]	0.03	864.96	1.29	0.20
Country of origin (reference: China)					
Germany	0.06 [0.00; 0.12]	0.03	849.33	1.92	0.06
France	-0.01 [-0.07; 0.05]	0.03	850.33	-0.25	0.80
Qualification (reference: vocational training)					
Graduate degree	0.01 [-0.04; 0.06]	0.03	865.05	0.42	0.68
Length of cooperation (reference: a few months)					
A few days	-0.09 [-0.14; -0.04]	0.03	864.79	-3.42	< 0.001

Note: β standardized regression coefficient; CI confidence interval; SD standard deviation; df degrees of freedom; t test statistic t; p probability

## Discussion

Our results indicate that nurses place the highest level of confidence in the skills of fictitious colleagues from Germany or spatially and culturally close countries to Germany like France in comparison to nurses from spatially and culturally more distant countries and health care systems like China. When it came to basic nursing skills like performing patient’s personal hygiene tasks, fictitious colleagues with professional training were more trusted than colleagues with academic training while the length of team collaboration was the main factor in trusting fictitious colleagues with specific nursing tasks like setting up an infusion. Finally, the age of fictitious colleagues and length of collaboration were associated with nurses’ willingness to swap shifts. Overall, these results require cautious interpretation and imply limitations in the derivation of practical implications since all effect sizes and respective confidence intervals are rather small with regard to the variance of the response scales, i.e., mostly under 0.5 on response scales ranging from one to six.

Study participants rated vignette scenarios similarly from their point of view and from the point of view of their actual colleagues which suggests that they assume that their actual team members would think and act similarly in the situations described. This finding might indicate that nurses share a perceived or actual common stock of knowledge for rules of interaction in everyday nursing care, e.g. with regard to trust in the competencies of their colleagues, the assessment of the skills of foreign-qualified nurses or the time required to be able to work together with new colleagues in a trusting manner. This orientation towards common standards indicates a high level of identification of the individual nursing staff with their own nursing team and the corresponding consequences for everyday nursing (collaboration) work. Nevertheless, the comparative consideration of the individual effect sizes in the evaluation of the factorial survey shows that it was primarily the participants’ own migration experience that was associated with their scenario assessment and comparatively less the diversity characteristics of the fictional colleagues. How nurses behave in direct interaction with colleagues might therefore mainly be determined by their own social group membership (e.g., having a history of migration) and less by the characteristics of the people with whom they interact. When comparing effect sizes, nurses’ own country of origin (as one category of social differentiation) or the one of the fictitious colleagues in vignettes in the scenarios of basic care and the setting up of an infusion is predominant. Thus, the following discussion mainly focusses on the topic of migration with a differentiation for the shift swapping (and partially for the infusion) scenario, where familiarity with team members seems paramount.

Differences in the assessment of colleagues’ skills depending on one’s own migration experience might stem from different sources. Qualitative studies found that academically qualified nurses who migrated to Germany show higher insensibility with the changes experienced in their professional identity and the required job profile in the new work environments, e.g. the need to carry out basic care activities [17, 18]. However, academically qualified nurses born in Germany reported to feel more comfortable to participate in all nursing tasks [18]. The stance on the provision of basic care activities, such as patient’s personal hygiene, is repeatedly reported as one of the distinctive features in the professional self-concept of nurses worldwide, where e.g. in some countries a more encompassing rather than diversified approach to patient care is taught and practiced [19]. A systematic review on the barriers and facilitators of integrating internationally qualified nurses in Australia also stresses foreign nurses’ challenges in adjusting their personal and professional identity and their understanding of the nursing role and professional relationships in the host country after migration [20]. Furthermore, a lack of perceived respect and trust in their abilities and knowledge by their team members was reported by migrated nurses [20]. These findings could partially explain our study results that nurses born in Germany are less sceptical when it comes to trust in the abilities of their colleagues’ skills in comparison to foreign-born nurses, who might be more likely to be less ready to trustfully interact with new team members due to their own experiences or due to their ongoing struggle to adapt to the work practices and work organization differing from those in their home country. In terms of Social Identity Theory, the results could also be interpreted as indicating that migrant nurses in Germany who work predominantly with colleagues without a migration background or with backgrounds from other countries might have less trust in these colleagues’ abilities because they constitute the out-group for these migrated nurses, e.g. because they initially might not share a common professional self-concept or understanding of how to perform certain nursing tasks.

Furthermore, our finding that nurses from France and especially China were less trusted with nursing activities than German nurses might stem from nurses’ acquired preconceptions or actual experiences with colleagues from these countries. France and China were used as proxies for countries which are considered to be on rather opposite sides of a spectrum of spatial and cultural proximity to Germany. Actual and alleged knowledge about and experience with the scope and quality of nursing training as well as the healthcare system in France might be more pronounced in German nurses in comparison to the same characteristics of nurses trained in China. Furthermore, nurses might draw conclusions about the skills of migrated nurses from their previous experiences with colleagues from other countries. The training of migrated nurses is usually time-consuming and challenging due to limited time and personnel resources on the ward and nurse managers as well as staff report missing support from their organizations in the integration and training of newly migrated nurses [18, 21]. This applies in particular to highly specified care environments such as intensive care units [17]. A lack of appropriate language skills by newly migrated nurses might be another reason for long-standing nurses to place less trust in the professional competencies of foreign-trained colleagues, especially in the beginning of work relationships, due to fear of incomplete or false communication [17, 21]. This is aggravated by the fact that nurses might be more focused on potential shortcomings in internationally trained nurses, e.g. with regard to basic care and their abilities and willingness to take up the majority “culture patterns”, than on these nurses’ present skills [18, 22]. However, other studies point out that nurses trained in the host country and nurse preceptors are aware of positive potential learning and reflecting opportunities from working with internationally trained colleagues, e.g. learning about the competencies of nurses from other countries and making use of the language skills in interactions with patients with limited German language skills [17, 21].

Other research shows that new members to closely knit teams were treated as “foreign bodies” to an organism, also with regard to stereotypes and bias against minority nurses. These teams tended to prefer new team members similar to them (e.g. with regard to ethnicity or migration background). However, prejudice and stereotypes persisted across different ethnic groups [23]. Another qualitative study conducted in Israel also revealed that minority nurses might prefer to work and interact with colleagues from the same national or religious background [24]. Research shows that lines of differentiation between team members and the creation of in- and outgroups is also drawn with regard to other personal characteristics such as nurses’ individual limitations in resilience due to chronic illnesses or limitations in flexibility due to family commitments or financial status [25]. Nonetheless, intersections of characteristics in the assessment of colleagues’ skills are likely to apply, e.g., middle-aged, non-migrated female nurses may represent the numerically largest group of nurses in a given ward and thus serve as the group that sets the ‘standards’ for judgment. In contrast, professional minorities in nursing, e.g., men, particularly young or elder colleagues, internationally qualified nurses, and university graduates, might be viewed and judged particularly critically by their colleagues. 

However, our findings suggest that trust in specific nursing skills like the setting up of an infusion is also affected by the familiarity with a team member. Thus, long-term collaboration between nurses, also daily ‘observation’ of colleagues’ work behaviors and skills, as suggested by Allport’s intergroup contact theory, might actually strengthen trust in the abilities of colleagues whom oneself not initially considers as part of the in-group. Similarly, nurses in our sample do not differentiate on the grounds of country of origin when it comes to collegial behavior, i.e. swapping shifts with a team member when being asked. Here, the familiarity with the respective colleague and an older age of the fictitious colleague were decisive in nurses’ attitudes, although there is an indication that nurses assume that country of origin might play a role in their actual colleagues’ decision in this situation. The slight preference for older nurses over middle-aged nurses in shift swapping might stem from different reasonings, e.g. a concession to the physical demands of the nursing profession especially for aging nurses or the status of older nurses as frequently established, reliable and respected members of a team. Thus, nurses’ prejudices might apply in the professional sphere regarding the assessment of their counterpart’s basic and treatment skills but length of cooperation might be the cornerstone of general teamwork skills and collegiality.

The practical relevance of the observed effects is difficult to assess within the scope of our factorial survey study. Since effect sizes are small and the sample is highly selective, practical implications should be considered with caution. However, in conjunction with other empirical studies on the challenges of team work in diverse nursing teams, the following deliberations are suggested as they target the whole system of nursing team work in hospitals with regard to organizationally and socially meaningful aspects instead of isolated factors based on individual statistical effect sizes from our study. Developing trusting relationships between team members in nursing might be achieved by various efforts. Diversity trainings are suggested in many publications to increase nurses’ and their leaders’ understanding of differences, e.g. between generations [26], international nursing qualification profiles [18] and moral emotions and professional values [22], and to identify avenues for the improvement of work environments and prevention or reduction of team conflict. Furthermore, nurses’ work conditions should allow for time in everyday work to engage in meaningful conversation and to share information and skills. Also, flexibility in working conditions in accordance with the diverse needs of a diverse workforce are demanded, e.g. regarding work hours and shift scheduling [26]. Especially when new nurses are trained, more organizational support to allow for sufficient time and personnel to familiarize new recruits with the work environment and professional tasks is demanded [17, 18, 21, 27].

### Limitations

Although we applied an innovative approach to study nurses’ perceptions in diverse work environments, our study is subject to limitations. First, all data is based on the subjective assessments of the participating nurses and can therefore not be objectified or verified in actual nursing practice. However, the original aim of the study was to investigate personal attitudes in the workplace, which legitimizes the use of self-report instruments. Second, due to the quantitative nature of the study and the associated requirements of calculable operationalizations of variables, a certain simplification of complex social dimensions of diversity is inevitable and respectively limits the insights into these topics. Third, the response rate of the online survey cannot be reliably determined due to a lack of information on the actual number of nurses reached by the various recruitment tools. If it is assumed that all of the approximately 11,400 nursing staff employed at both institutions learned about the study through emails, intranet messages and flyers, the response rate is only around 6% with 684 final participants. No data are available regarding the sociodemographic characteristics of the nursing personnel employed in the two institutions included in this study. However, the limited national statistics on nursing staff in Germany indicate that 82% of nurses are female, 82% possess German citizenship, 78% of nurses working in hospitals are qualified nursing professionals, and approximately two thirds are between 25 and 54 years of age [1]. Thus, our sample includes slightly more male nurses, more nurses with a German citizenship acquired by birth, more nurses aged 25 to 54 years, and more nurses with at least vocational training. Taking into account the presumably low participation rate, the specific sampling approach at two healthcare organizations in a major German city, and the overrepresentation of nurses with specific demographic backgrounds, it cannot be assumed that the results of this study are representative of all nurses working in Germany. Thus, selection bias might have occurred such that perspectives of female nurses with a migration background, specifically younger and older nurses, and those with a nursing assistant training or university degree are underrepresented in our sample. Finally, although participation in the survey was voluntary and anonymously, and participants’ answers to vignettes from their point of view only slightly differed from those answers from their actual colleagues’ point of view, social desirability bias in response behavior to vignettes cannot be completely ruled out.

## Conclusions

Nurses’ assessments of their colleagues’ professional abilities and their personal willingness to swap shifts is most strongly associated with their own social group membership, mainly their migration experience, and less strongly by the diversity characteristics of their counterparts. The factorial survey method permits the experimental examination of the relevance of multiple dimensions of diversity by simultaneously incorporating various dimensions of variables in statistical analyses. Nevertheless, future quantitative research on contributing factors for successful teamwork should investigate additional potentially relevant diversity dimensions, such as religion, social class or disability with sufficiently large sample sizes, and apply more complex interaction terms in addition to direct effects to approximate the implications of research on intersectionality and postulates of SIT, e.g. considering the effects of respondents’ and the fictitious persons’ convergence in social categories. Furthermore, mixed methods research is needed to understand the intra- and interpersonal as well as organizational trajectories in the development of trust in each other’s capabilities and the prerequisites for cooperation in German nursing teams, especially under the conditions of the prevailing shortage of skilled nurses and the challenges posed by the demographic change. Although our study findings from a highly selective sample only permit cautious implications for clinical practice, we deem that a comprehensive and structured onboarding of new team members could contribute to solid and sustainable working relationships from the beginning of team formation and development. To further improve existing and future work relationships in nursing teams, dedicated team trainings and changes in work organization with the aim to increase professional and personal exchange between team members might promote mutual understanding, trust, learning and cooperation, which in turn has the potential to improve patient care.

## Supplementary Information

Below is the link to the electronic supplementary material.

Supplementary Material 1

## Data Availability

Due to restrictions defined in the approved ethics application for this study, the datasets generated and analyzed during this study cannot be shared with third parties. Data sharing is therefore not permitted.

## Abbreviations

EU: European Union
QR: Quick-response
SD: Standard deviation
