# Supplementary material for: How are nurses’ individual and their colleagues’ diversity factors associated with their cooperation behaviors in teams? - Results of an online factorial survey
Source: BMC Nurs. 2026 Mar 24;25:336. doi: 10.1186/s12912-026-04572-5 (PMC13063756; doi:10.1186/s12912-026-04572-5)
Supplement: Supplementary file 1 — Supplementary Material 1 [file 12912_2026_4572_MOESM1_ESM.docx]

## Supplemental material 1: English translation of the case scenarios 1-3

## Case scenario 1: "Patient’s personal hygiene"

| You are on the early shift, and your team is fully staffed. You are responsible for a female patient for whom you plan to carry out personal hygiene next. However, due to a new admission, you are unable to perform this task yourself. It so happens that your 24-year-old \| 42-year-old \| 59-year-old *(dimension: age)* colleague^[[1]](#footnote-1)^ M. *(dimension: gender)*, who comes from Germany \| France \| China *(dimension: country of origin)* and has studied nursing \| completed nursing training there *(dimension: qualification)*, contacts you and offers to take over the patient’s personal hygiene on your behalf. | | | | | | |
| --- | --- | --- | --- | --- | --- | --- |
| **I have confidence in M.’s ability to perform the patient’s personal hygiene.**  Please indicate on a scale from 1 (“Does not apply at all”) to 6 (“Applies fully”) the extent to which the above statement applies. | | | | | | |
| Does not apply at all | Does not apply | Somewhat does not apply | Somewhat applies | Applies | Applies fully | No response |
| ☐  1 | ☐  2 | ☐  3 | ☐  4 | ☐  5 | ☐  6 | ☐ |
|  |  |  |  |  |  |  |
| Please think about your current nursing team.  *If you are currently working in several teams, please refer to the nursing team in which you most recently worked.*  **Colleagues in my work environment would have confidence in M.’s ability to perform the patient’s personal hygiene.** | | | | | | |
| Does not apply at all | Does not apply | Somewhat does not apply | Somewhat applies | Applies | Applies fully | No response |
| ☐  1 | ☐  2 | ☐  3 | ☐  4 | ☐  5 | ☐  6 | ☐ |

| **How realistic is this situation in your daily work routine?** | | | | | | |
| --- | --- | --- | --- | --- | --- | --- |
| Not at all realistic | Not realistic | Rather not realistic | Somewhat realistic | Realistic | Very realistic | No response |
| ☐  1 | ☐  2 | ☐  3 | ☐  4 | ☐  5 | ☐  6 | ☐ |

## Case scenario 2: “Starting an infusion”

| You are on the early shift, and your team is fully staffed. Shortly before your breakfast break, you remember that you need to administer an infusion to one of your patients. In the hallway, you start a conversation with your 24-year-old \| 42-year-old \| 59-year-old *(dimension: age)* colleague^1^ A. *(dimension: gender)*. A. offers to prepare and administer the infusion on your behalf, as he/she has just finished his/her break. Colleague A. studied nursing \| completed nursing training *(dimension: qualification)* in Germany \| France \| China *(dimension: country of origin)* and has been working with you in the team for a few days \| several months *(dimension: length of collaboration)*. | | | | | | |
| --- | --- | --- | --- | --- | --- | --- |
| **I have confidence in A.’s ability to prepare and administer the infusion.**  Please indicate on a scale from 1 (“Does not apply at all”) to 6 (“Applies fully”) the extent to which the above statement applies. | | | | | | |
| Does not apply at all | Does not apply | Somewhat does not apply | Somewhat applies | Applies | Applies fully | No response |
| ☐  1 | ☐  2 | ☐  3 | ☐  4 | ☐  5 | ☐  6 | ☐ |
|  |  |  |  |  |  |  |
| Please think about your current nursing team.  *If you are currently working in several teams, please refer to the nursing team in which you most recently worked.*  **Colleagues in my work environment would have confidence in A.’s ability to prepare and administer the infusion.** | | | | | | |
| Does not apply at all | Does not apply | Somewhat does not apply | Somewhat applies | Applies | Applies fully | No response |
| ☐  1 | ☐  2 | ☐  3 | ☐  4 | ☐  5 | ☐  6 | ☐ |

| **How realistic is this situation in your daily work routine?** | | | | | | |
| --- | --- | --- | --- | --- | --- | --- |
| Not at all realistic | Not realistic | Rather not realistic | Somewhat realistic | Realistic | Very realistic | No response |
| ☐  1 | ☐  2 | ☐  3 | ☐  4 | ☐  5 | ☐  6 | ☐ |

## Case scenario 3: “Swapping shifts”

| Shortly before leaving the ward or unit at the end of your shift, your 24-year-old \| 42-year-old \| 59-year-old *(dimension: age)* colleague^1^ W. *(dimension: gender)* approaches you. W. asks whether you could swap shifts next Sunday. Because W. has an urgent appointment, he/she is hoping for your help. On that particular day, you have no personal plans yourself. You have been working with W. for a few days \| several months *(dimension: length of collaboration)*. W. comes from Germany \| France \| China *(dimension: country of origin)* and has studied nursing \| completed nursing training *(dimension: qualification)*. | | | | | | |
| --- | --- | --- | --- | --- | --- | --- |
| **I am willing to swap shifts with W.**  Please indicate on a scale from 1 (“Does not apply at all”) to 6 (“Applies fully”) the extent to which the above statement applies. | | | | | | |
| Does not apply at all | Does not apply | Somewhat does not apply | Somewhat applies | Applies | Applies fully | No response |
| ☐  1 | ☐  2 | ☐  3 | ☐  4 | ☐  5 | ☐  6 | ☐ |
|  |  |  |  |  |  |  |
| Please think about your current nursing team.  *If you are currently working in several teams, please refer to the nursing team in which you most recently worked.*  **Colleagues in my work environment would be willing to swap shifts with W.** | | | | | | |
| Does not apply at all | Does not apply | Somewhat does not apply | Somewhat applies | Applies | Applies fully | No response |
| ☐  1 | ☐  2 | ☐  3 | ☐  4 | ☐  5 | ☐  6 | ☐ |

| **How realistic is this situation in your daily work routine?** | | | | | | |
| --- | --- | --- | --- | --- | --- | --- |
| Not at all realistic | Not realistic | Rather not realistic | Somewhat realistic | Realistic | Very realistic | No response |
| ☐  1 | ☐  2 | ☐  3 | ☐  4 | ☐  5 | ☐  6 | ☐ |

Supplemental Material 2: Results of the random intercept model on the outcome “confidence in colleagues’ competencies regarding patient’s personal hygiene” from colleague’s perspective

| Parameter | Estimate  [95% CI] | SD | df | T | p-level |
| --- | --- | --- | --- | --- | --- |
| Constant term | 4.36  [3.97; 4.72] | 0.19 | 471.25 | 22.70 | <.001 |
| Participant variables (macro level) |  |  |  |  |  |
| Gender (reference: female) |  |  |  |  |  |
| male | 0.07  [-0.13; 0.26] | 0.10 | 442.31 | 0.69 | .49 |
| diverse | 0.19  [-0.58; 0.97] | 0.39 | 441.44 | 0.49 | .62 |
| Age | 0.01  [0.00; 0.01] | 0.00 | 443.64 | 1.54 | .12 |
| Birth country (reference: outside Germany) |  |  |  |  |  |
| Germany | 0.40  [0.13; 0.67] | 0.14 | 443.57 | 2.89 | .004 |
| Qualification (reference: nurse graduate) |  |  |  |  |  |
| Nursing assistant | -0.14  [-0.57; 0.29] | 0.22 | 442.25 | -0.66 | .51 |
| Registered nurse | -0.05  [-0.27; 0.17] | 0.11 | 445.08 | -0.42 | .68 |
| Vignette variables (micro level) |  |  |  |  |  |
| Age (reference: 59 years) |  |  |  |  |  |
| 24 years | -0.02  [-0.08; 0.05] | 0.03 | 878.35 | -0.55 | .58 |
| 42 years | 0.03  [-0.04; 0.10] | 0.03 | 880.41 | 0.92 | .36 |
| Gender (reference: female) |  |  |  |  |  |
| Male | -0.02  [-0.08; 0.04] | 0.03 | 903.45 | -0.71 | .48 |
| Country of origin (reference: China) |  |  |  |  |  |
| Germany | 0.34  [0.27; 0.40] | 0.03 | 879.65 | 10.06 | <.001 |
| France | 0.16  [0.09; 0.23] | 0.03 | 878.63 | 4.74 | <.001 |
| Qualification (reference: vocational training) |  |  |  |  |  |
| Graduate degree | -0.04  [-0.09; 0.02] | 0.03 | 903.69 | -1.27 | .20 |

Supplemental Material 3: Results of the random intercept model on the outcome “confidence in colleagues’ competencies regarding infusion insertion” from colleague’s perspective

| Parameter | Estimate [95% CI] | SD | df | T | p-level |
| --- | --- | --- | --- | --- | --- |
| Constant term | 4.36  [3.87; 4.85] | 0.25 | 454.44 | 17.57 | <.001 |
| Participant variables (macro level) |  |  |  |  |  |
| Gender (reference: female) |  |  |  |  |  |
| Male | 0.14  [-0.11; 0.39] | 0.13 | 408.77 | 1.13 | .26 |
| Diverse | 0.28  [-0.66; 1.21] | 0.48 | 403.43 | 0.58 | .56 |
| Age | 0.00  [-0.01; 0.01] | 0.01 | 409.85 | -0.25 | .80 |
| Birth country (reference: outside Germany) |  |  |  |  |  |
| Germany | 0.31  [-0.04; 0.66] | 0.18 | 403.64 | 1.75 | .08 |
| Qualification (reference: nurse graduate) |  |  |  |  |  |
| Registered nurse | 0.10  [-0.18; 0.38] | 0.14 | 403.99 | 0.70 | .48 |
| Vignette variables (micro level) |  |  |  |  |  |
| Age (reference: 59 years) |  |  |  |  |  |
| 24 years | 0.01  [-0.10; 0.11] | 0.05 | 788.50 | 0.14 | .89 |
| 42 years | -0.06  [-0.16; 0.04] | 0.05 | 788.03 | -1.13 | .26 |
| Gender (reference: female) |  |  |  |  |  |
| Male | -0.04  [-0.13; 0.05] | 0.05 | 820.83 | -0.89 | .37 |
| Country of origin (reference: China) |  |  |  |  |  |
| Germany | 0.48  [0.38; 0.58] | 0.05 | 787.83 | 9.20 | <.001 |
| France | 0.20  [0.10; 0.30] | 0.05 | 789.24 | 3.80 | <.001 |
| Qualification (reference: vocational training) |  |  |  |  |  |
| Graduate degree | 0.02  [-0.07; 0.11] | 0.05 | 819.75 | 0.39 | .70 |
| Length cooperation (reference: a few months) |  |  |  |  |  |
| A few days | -0.44  [-0.53; -0.35] | 0.05 | 820.78 | -9.85 | <.001 |

Supplemental Material 4: Results of the random intercept model on the outcome “willingness to swap shifts” from colleague’s perspective

| Parameter | Estimate | SD | df | T | p-level |
| --- | --- | --- | --- | --- | --- |
| Constant term | 4.46  [4.04; 4.87] | 0.21 | 449.52 | 21.01 | <.001 |
| Participant variables (macro level) |  |  |  |  |  |
| Gender (reference: female) |  |  |  |  |  |
| Male | 0.09  [-0.13; 0.30] | 0.11 | 421.06 | 0.78 | .44 |
| Diverse | -0.31  [-1.14; 0.52] | 0.42 | 420.16 | -0.74 | .46 |
| Age | 0.00  [-0.01; 0.01] | 0.00 | 421.86 | 0.19 | .85 |
| Birth country (reference: outside Germany) |  |  |  |  |  |
| Germany | 0.25  [-0.05; 0.55] | 0.15 | 425.38 | 1.66 | .10 |
| Qualification (reference: nurse graduate) |  |  |  |  |  |
| Nursing assistant | -0.24  [-1.56; 1.08] | 0.67 | 420.28 | -0.35 | .73 |
| Registered nurse | 0.12  [-0.12; 0.36] | 0.12 | 423.50 | 0.98 | .33 |
| Vignette variables (micro level) |  |  |  |  |  |
| Age (reference: 59 years) |  |  |  |  |  |
| 24 years | -0.01  [-0.07; 0.06] | 0.03 | 834.55 | -0.14 | .89 |
| 42 years | -0.03  [-0.10; 0.04] | 0.03 | 835.25 | -0.91 | .36 |
| Gender (reference: female) |  |  |  |  |  |
| Male | 0.04  [-0.02; 0.09] | 0.03 | 854.93 | 1.17 | .24 |
| Country of origin (reference: China) |  |  |  |  |  |
| Germany | 0.11  [0.05; 0.18] | 0.03 | 834.97 | 3.33 | <.001 |
| France | -0.02  [-0.09; 0.04] | 0.03 | 835.08 | -0.68 | .50 |
| Qualification (reference: vocational training) |  |  |  |  |  |
| Graduate degree | -0.01  [-0.07; 0.05] | 0.03 | 855.12 | -0.25 | 0.80 |
| Length cooperation (reference: a few months) |  |  |  |  |  |
| A few days | -0.08  [-0.14; -0.02] | 0.03 | 854.84 | -2.66 | .01 |

1. In the German language, many nouns, especially terms for people and professions, are used in a grammatically masculine or feminine form, e.g. the noun „colleague“ *[Kollege/Kollegin]*. [↑](#footnote-ref-1)
